# Back to basics: a rare and aggressive case of *Aggregatibacter aphrophilus* endocarditis

**DOI:** 10.1093/omcr/omab043

**Published:** 2021-06-18

**Authors:** Nada Al-Sakini, Charo Bruce, Samuel Seitler, Wasyla Ibrahim, Victoria Nicholas, Orphelie Loup, Darryl Shore, Wei Li, Michael A Gatzoulis

**Affiliations:** Department of Cardiology, Adult Congenital Heart Centre and National Centre for Pulmonary Hypertension, Royal Brompton & Harefield NHS Trust, National Heart & Lung Institute, Imperial College, London, UK

**Keywords:** Cardiothoracic Surgery

## Abstract

We present the case of a 25-year-old with a history of bicuspid aortic valve and ascending aortopathy who was successfully treated for infective endocarditis (IE) due to *Aggregatibacter aphrophilus*. His clinical course was complicated by a large aortic root abscess not initially visualised on transthoracic echocardiography or computerised tomography. The cardinal feature of progressive prolongation of the PR interval on serial electrocardiograms was the only sign associated with clinical deterioration and was the trigger for rapid investigation and urgent management. Our case emphasises the importance of simple bedside tests to identify dynamic clinical scenarios and the requirement for early further imaging in the management of IE.

## INTRODUCTION

The Haemophilus, Aggregatibacter, Cardiobacterium, Eikenella, Kingella (HACEK) group is a collection of gram-negative coccobacillary organisms which are components of the normal flora of the oropharynx. *Aggregatibacter aphrophilus* is a member of the HACEK group; a haemophilus species. It is a rare cause of infective endocarditis (IE), more commonly seen in patients with immunosuppression, congenital heart disease and trauma [1]. We present a case of a rapidly evolving catastrophic aortic root abscess following *A. aphrophilus* infection, initially suspected following progressive electrocardiogram (ECG) changes. This case emphasises the simple tests every doctor must be cognisant of, especially when the diagnosis is unclear.

## CASE REPORT

A 25-year-old male was admitted for elective aortic and pulmonary valve replacement. On admission, he was found to have a C-reactive protein (CRP) of 102 mg/l with normal white cell count. His past medical history was significant for congenital heart disease with a true bicuspid aortic valve and ascending aortopathy. He was asymptomatic as a child and remained under surveillance for gradual worsening of his aortic stenosis until undergoing balloon dilatation aged 9, followed by a Ross procedure aged 15 for severe stenosis. He developed progressive aortic root dilation and underwent a redo operation with a Bentall procedure using a 29-mm Perimount prosthesis aged 20. He remained asymptomatic over the following years, until recent echocardiographic surveillance showed development of severe aortic stenosis and mixed disease of the right ventricle–pulmonary artery (RV–PA) homograft, prompting admission for elective surgical intervention.

**Figure 1:**
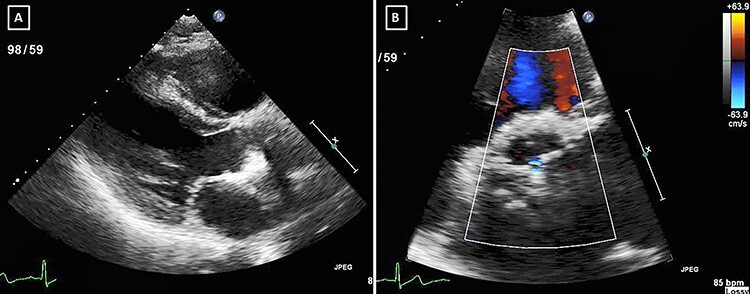
Initial TTE showing no evidence of para-aortic collection (**A**) parasternal long-axis view, (**B**) short-axis view with colour Doppler.

**Figure 2:**
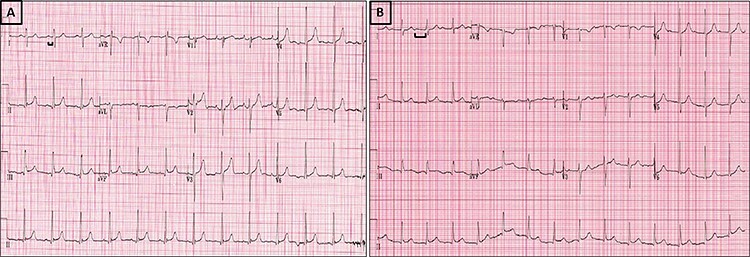
ECGs showing progressive significant increase in PR interval on admission (**A**) PR = 158 ms, compared with Day 5 (**B**) increased to 292 ms.

**Figure 3:**
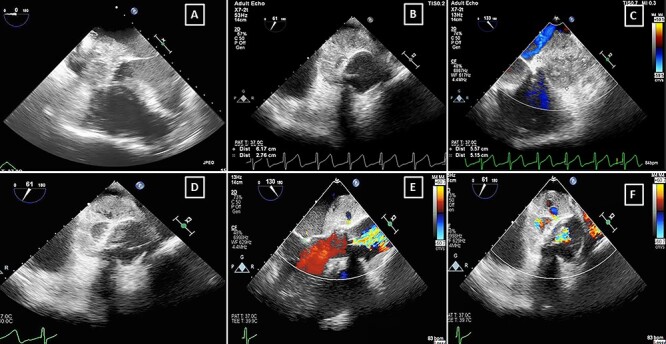
TOE showing the large vacuolated aortic root abscess (**A**) four-chamber view, (**B**) at 61°, (**C**) 133° with colour Doppler, (**D**) 61° short axis, (**E**) 130° short axis with colour Doppler showing systolic flow through the abscess indicating fistula formation with the aortic root, (**F**) 61° Short axis with colour Doppler showing the communication of the aortic root abscess to the aorticroot.

On admission the patient reported to feeling well, aside from recent flu-like illness which had resolved a week prior. At that time, he had been feeling feverish with myalgia and a non-productive cough. He denied any localising symptoms and his observations were all within normal limits. His physical examination was normal, barring the expected ejection systolic and early diastolic murmurs, with no peripheral stigmata of endocarditis. On the day of admission, he underwent a computerised tomography (CT) scan of the aorta in preparation for surgery, which included views of the chest and abdomen. Review of this scan revealed no focus of infection, or para-aortic collections. His urine dipstick and respiratory viral PCR screen were both negative.

While awaiting surgery, he began to clinically deteriorate with tachycardia and fevers. Repeated transthoracic echocardiography (TTE) showed no obvious independently mobile structures on the valves, however due to the extent of degenerative changes of the valve it was difficult to fully exclude vegetations (Fig. 1). The patient’s CRP continued to rise over the next 2 days and his blood cultures came back positive for *A. aphrophilus*.

**Figure 4:**
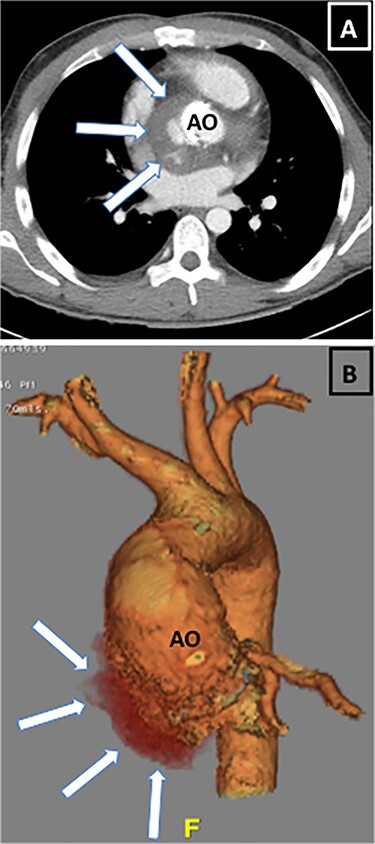
White arrows on CT showing expanding aortic root abscess (**A**) short-axis view, (**B**) rendered reconstruction. AO = Aorta.

He was started on Ceftriaxone and Gentamycin, however by Day 5 his CRP had climbed to 317 mg/l with a white cell count of 12.8 × 10^9^ g/l and he remained pyrexial. Most significantly, his ECGs showed progressive prolongation of PR interval (Fig. 2) from a baseline of 158 to 292 ms. The daily progressive PR interval prolongation was highly suspicious of aortic root abscess formation and triggered urgent further imaging. Transoesophageal echocardiography (TOE) showed a large echo-dense structure around the aortic root extending posteriorly towards the left atrium. Several small cavities were seen in the mass with blood flow and communication between the cavity and the aortic root (Fig. 3A–F, Videos 1–4). There was also localised extension of the abscess border into the left atrium with potential risk of rupture. Repeat CT was carried out which showed an increase in low attenuation material around the aortic root with two new regions of internal contrast extravasation. This was in keeping with aortic root abscess at the level of the aortic prosthesis with extension into the roof of the left atrium (Fig. 4).

He was taken urgently to theatre for debridement and redo-Bentall operation with a mechanical aortic valve and replacement of the RV to PA conduit. Explanted tissue did not grow anything at culture, but 16S rDNA PCR screen was positive and seq1 PCR detected *A. aphrophilus.* He had an uncomplicated recovery post-operatively and was treated with a further 2 weeks of intravenous antibiotics with appropriate normalisation of his CRP. Antibiotic treatment was changed to oral Ciprofloxacin and completed a total of course of 6 weeks. He remained well since stopping antibiotics with no further fevers and normalCRP.

## DISCUSSION

This case highlights the importance of basic tests and investigations in the management of IE. Daily ECGs in IE are often overlooked but progressive PR prolongation was an early sign of worsening underlying pathology and a trigger for urgent action in this case. Aortic root abscesses are a rare but life-threatening complication of IE, predominantly associated with aortic valve endocarditis [2]. PR prolongation occurs with aortic root abscesses due to the proximity of the cardiac conduction system. The atrioventricular node sits at the base of the atrial septum with the non-penetrating His bundle travelling along the membranous septum to become the penetrating bundle, passing though the central fibrous body at the apex of the triangle of Koch and directly into the left ventricular outflow tract [3]. This pathway takes the conduction system directly beneath the commissure between the non-coronary and right coronary aortic leaflets, as well as in close relation to the mitral and tricuspid valves.

The importance of taking multiple blood cultures is also highlighted. Our patient had five sets of blood cultures before being started on antibiotic therapy, two of which were negative at 7 days of incubation. The HACEK group is notoriously difficult to culture and often require prolonged incubation or analysis using DNA PCR [4]. The likelihood of growing organisms is further reduced following antibiotic therapy therefore at least three cultures should be taken for suspected IE patients before treatment as per European Society of Cardiology guidelines [5].

The case emphasises the value of the fundamental investigations which underpin the management of IE, in particular serial blood cultures and daily ECGs. These crucial tests help guide therapy and are important predictors of evolving complications. This case also demonstrates the value of multi-modality imaging in endocarditis and the importance selecting different imaging modalities when the clinical picture alters, particularly in high-risk groups such as congenital heart disease patients.

## CONFLICTS OF INTEREST STATEMENT

All authors declare no conflicts of interest.

## FUNDING

No sources of funding were required.

## ETHICAL APPROVAL

No ethical approval was required.

## CONSENT

Written consent for submission and publication of this case report including images and associated text has been obtained from the patient.

## GUARANTOR

The nominated Guarantor is Dr Nada Al-Sakini. This manuscript has not been published and is not under consideration for publication elsewhere. All the authors have read the manuscript and have approved this submission.

## Supplementary Material

Video_2_omab043

Video_3_omab043

Video_4_omab043

Video_5_omab043
